# A Rare Case of Unresectable Adenoid Cystic Carcinoma of the Nasopharynx Treated with Intensity Modulated Proton Therapy

**DOI:** 10.7759/cureus.1688

**Published:** 2017-09-15

**Authors:** Jae Phan, Sweet Ping Ng, Courtney Pollard, Jack Phan

**Affiliations:** 1 Department of Neuroscience, Rice University; 2 Department of Radiation Oncology, The University of Texas MD Anderson Cancer Center

**Keywords:** adenoid cystic carcinoma, impt, radiation oncology, proton therapy

## Abstract

Adenoid cystic carcinoma (ACC) is generally treated with surgical resection followed by postoperative radiotherapy. In cases where surgical management is precluded due to the location of the tumor and/or patient factors, radiation therapy can be offered to achieve local control. Here, we present a case of unresectable Stage T4N0 ACC of the nasopharynx with skull base and intracranial extension treated with intensity modulated proton therapy (IMPT), which achieved good local control with no significant late toxicity.

## Introduction

Adenoid cystic carcinoma (ACC) is a rare type of cancer that possesses an indolent growth rate despite** **having** **a relentless course with a high rate of recurrence. ACC arises within the major and minor secretory glands of the head and neck and is known for its tendency to invade the peripheral nerves [1-2]. Unlike most carcinomas, this disease seldom metastasizes to regional lymph nodes but can metastasize to distant sites with the lungs being the most common location [1]. For patients with locally advanced ACC, surgical resection is considered to be the standard form of treatment. However, for** **caseswhere surgery is not feasible, definitive radiation may be offered to provide local disease control. In patients with ACC of the skull base, with or without intracranial extension, surgery is usually not feasible due to the difficulty in achieving negative margins. Delivering definitive radiation to the disease in the skull base can be a challenge secondary to the close proximity of radiosensitive normal structures. The treating radiation oncologist must carefully balance the delivery of a tumoricidal radiation dose while meeting the dose constraints to normal structures, as both the local progression of disease and the development of radiation-related toxicities can cause significant morbidity to the patient.** **With advances in image-guided radiotherapy (IGRT) and other highly conformal techniques, such as intensity modulated proton therapy (IMPT), higher doses of radiation can be delivered with a lower risk of developing severe acute and late toxicities. Here, we present a rare case of unresectable stage T4N0 ACC of the nasopharynx treated with IMPT. Informed consent was obtained from the patient for this study.

## Case presentation

**Figure 1:**
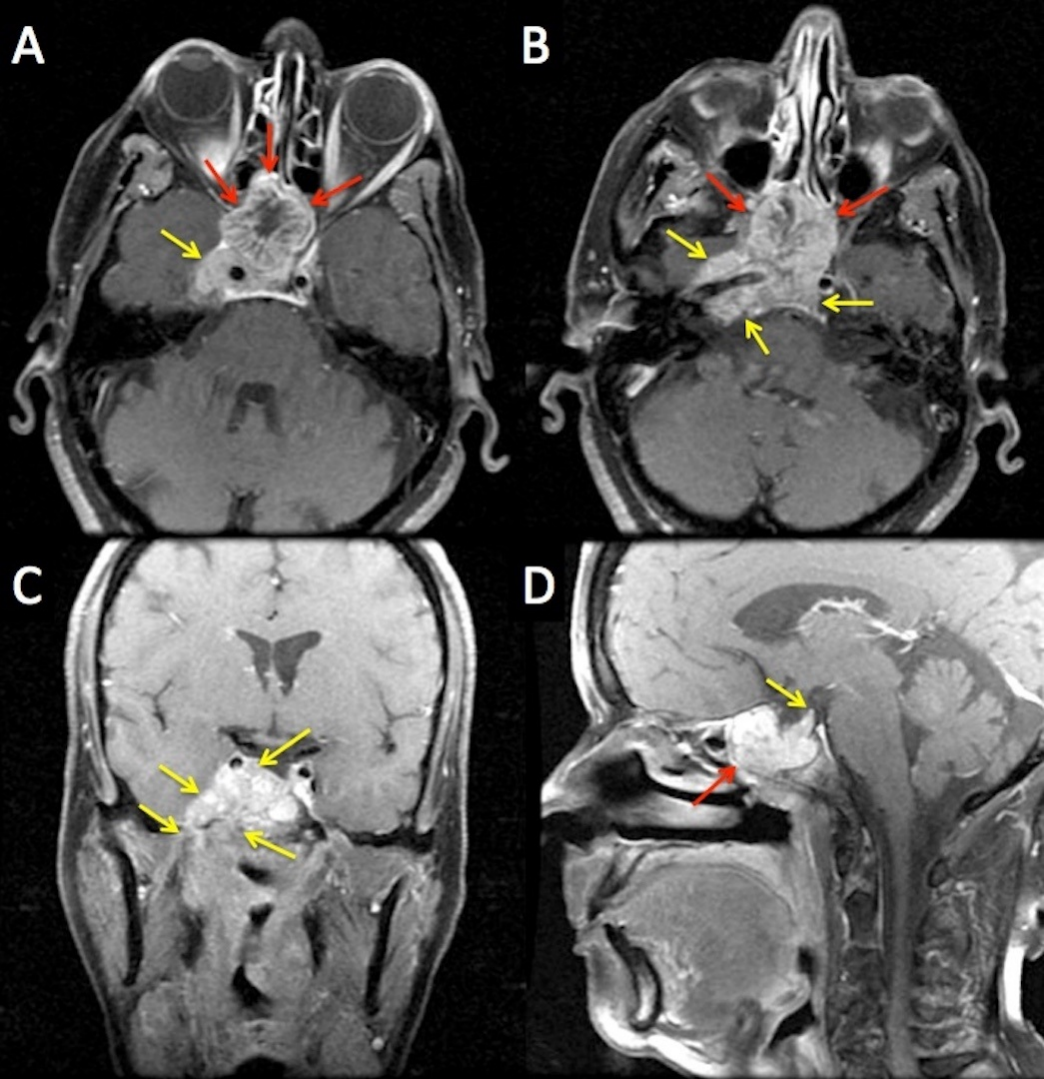
Representative MRI images at diagnosis, showing the large nasopharyngeal mass with intracranial extension Large, exophytic, primary tumor (red and yellow arrows) in nasopharynx (red arrows) invading into the right foramen ovale and the cavernous sinus (yellow arrows). The tumor is in close proximity to the normal brain, optic nerve, optic chiasm, and globes. A and B - axial images showing the nasopharyngeal mass (red arrows) and the intracranial extent into the cavernous sinus and the proximity of the tumor to the brainstem (yellow arrows), C - coronal image showing the superior and inferior extent of the intracranial portion of the tumor (yellow arrows), D - sagittal image showing tumor involvement of the clivus (red arrow) and the intracranial extent of the tumor, in proximity to the brain stem (yellow arrow). MRI: Magnetic resonance imaging

A 71-year-old female presented with a three-year history of progressive headaches and a feeling of 'pressure' in her right ear. Her symptoms worsened over a year and she saw an ear, nose, and throat (ENT) surgeon who placed a tympanostomy tube in her right ear. When this procedure failed to relieve her symptoms, a nasoendoscopy was performed, revealing a large protruding mass on the right side of the nasopharynx. A computed tomography (CT) scan revealed a large lesion in the nasopharynx and the posterior sphenoid sinus. This was further characterized by a magnetic resonance imaging (MRI) scan and a positron emission tomography (PET) scan. The MRI showed a 4.4 x 3.8 x 5 cm tumor centered within the sphenoid sinus, extending into the right cavernous sinus (Figure 1). There was tumor expansion into the right foramen ovale and involvement of cranial nerve (CN) V3 into the masticator space. Inferiorly, the tumor extended from the nasopharynx towards the right jugular foramen. The PET scan showed a fluorodeoxyglucose (FDG) –avid sphenoid sinus tumor and confirmed the local extent of the disease with no evidence of regional or distant metastases. The patient underwent a biopsy, which confirmed adenoid cystic carcinoma (ACC). This patient was staged as having a T4N0 ACC of the nasopharynx.

Due to the location of the lesion, it was determined to be unresectable by the surgical team. Definitive radiotherapy with concurrent chemotherapy was recommended after discussion at the institutional multidisciplinary tumor board. The patient was planned to be treated with intensity modulated proton therapy (IMPT) to a dose of 6996 cGe in 33 fractions over 6.5 weeks with concurrent chemotherapy (six weekly cycles of cisplatin). The patient was brought to the simulation suite and immobilized using a thermoplastic mask, proton-compatible immobilization bag, and an oral stent. CT images obtained in the treatment position were transferred to the Eclipse treatment planning system (Varian Medical System, Palo Alto, CA). Planning CT images were also fused with the MRIs obtained in the treatment position to assist with accurate target delineation. Given the predilection of ACC for perineural spread, the involved cranial nerve (CN V3) was tracked and targeted up to the skull base (foramen ovale). 

**Figure 2:**
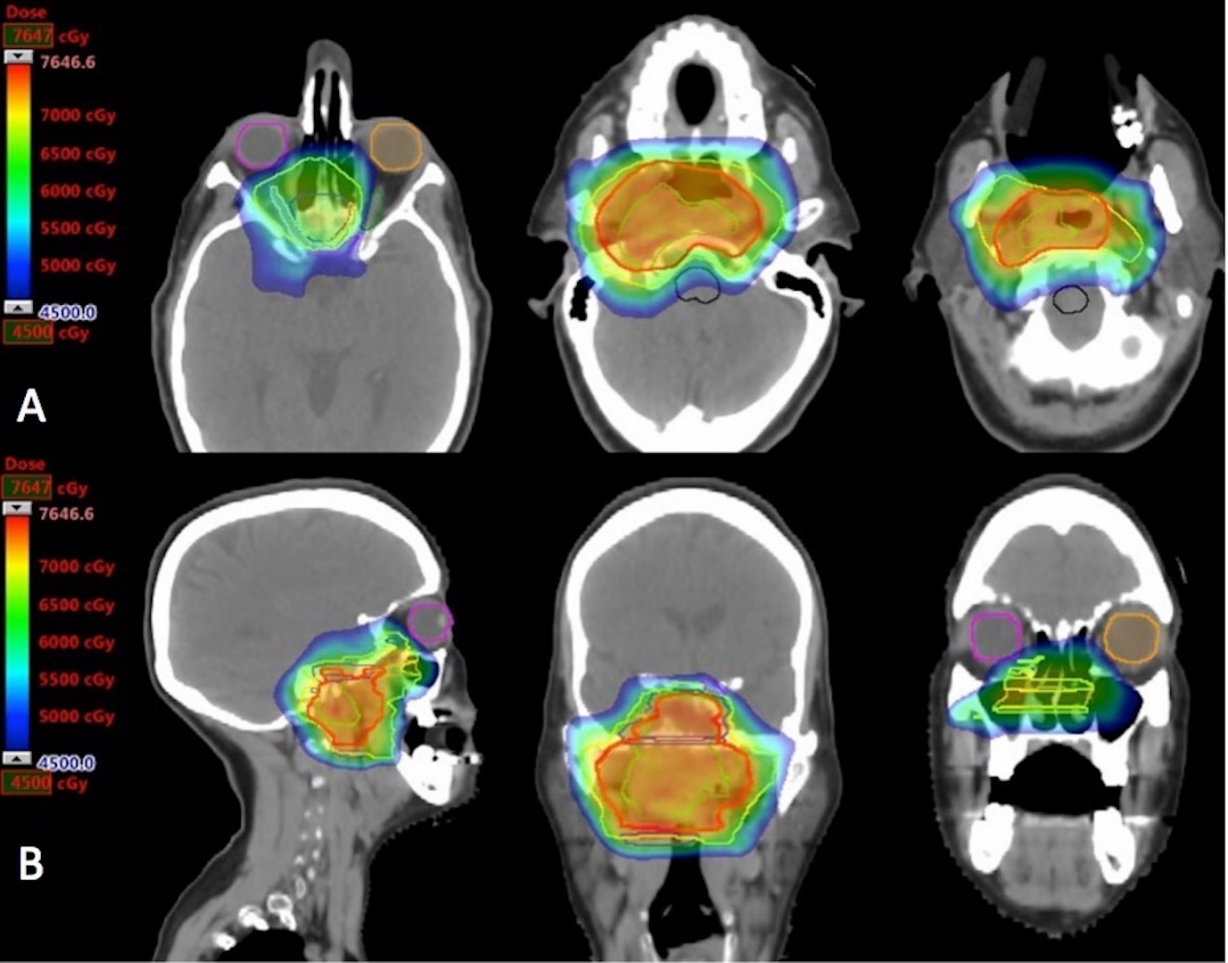
Intensity modulated proton therapy plan Images in row A are representative axial images of the treatment plan. Images in row B are representative sagittal (left) and coronal (middle and right) images of the treatment plan. Gross disease is covered in the high dose cloud (red, 6996 cGE). The yellow dose cloud represents coverage of microscopic disease spread (6300 cGE). Images demonstrate rapid dose fall off of proton beam therapy in relation to the intended target. Organs at risk shown here are right eye (purple), left eye (orange), right optic nerve (light blue), left optic nerve (dark green), and brainstem (black).

**Figure 3:**
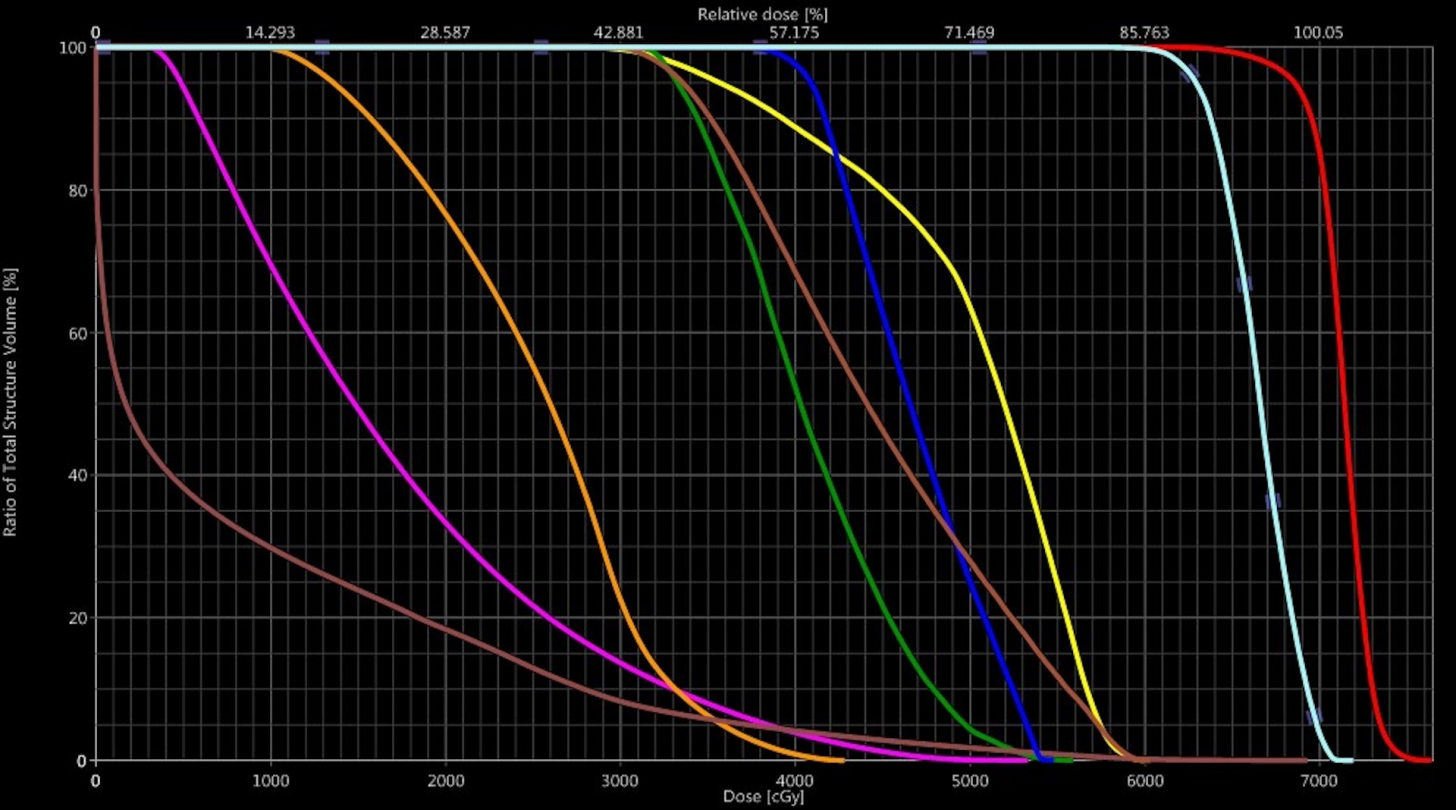
Dose volume histogram (DVH) DVH showing some of the doses to target regions (clinical target volumes 70 Gy in red and 63 Gy in cyan) and critical structures (right eye - pink, right optic nerve - yellow, left eye - orange, left optic nerve - green, whole brain - brown, optic chiasm - blue, brain stem - light brown)

Due to the complexity of the patient's tumor location in close proximity to critical normal structures, plans for both photon-based intensity modulated radiation therapy (IMRT) and IMPT were generated to compare the dose distributions to the target and to the organs at risk. Ultimately, the proton therapy plan was chosen because it delivered lower doses to nearby critical neural and optic structures without compromising target coverage. The final IMPT treatment plan is shown in Figure 2, with the resultant minimal dose to the adjacent normal brain, spinal cord, optic nerves, optic chiasm, and oral cavity. Additionally, a dose volume histogram (DVH) is provided, showing doses to the target region, adjacent optic apparatus, and neural critical structures (Figure 3). 

The patient tolerated her radiation treatment well and completed treatment as scheduled, without breaks. By the end of radiotherapy, she had Grade 2 dysgeusia, Grade 3 mucositis, and Grade 2 dermatitis.

**Figure 4:**
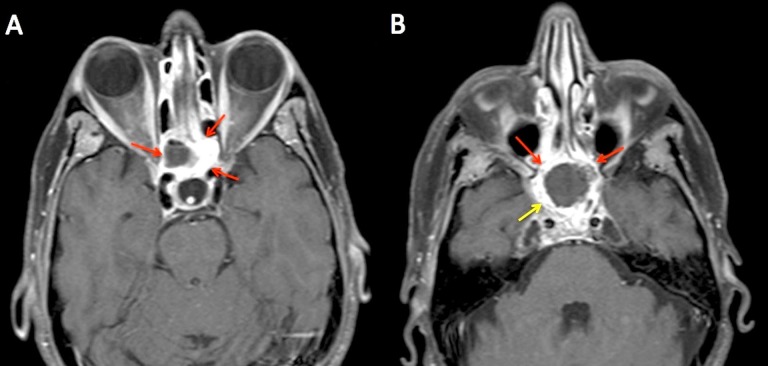
Representative axial slices of post-treatment MRI obtained at four months after completion of treatment Significant regression of the large nasopharyngeal mass and disease in the cavernous sinus (red and yellow arrows). Image A - the nasopharyngeal portion of the tumor had regressed significantly with residual cystic mass (red arrows). Image B - superior portion of the tumor was largely cystic (red arrows) and previous intracranial disease (yellow arrow) had regressed. MRI: Magnetic resonance imaging

At her four-month follow-up visit, the patient had a good response to treatment with a post-treatment MRI showing a significant decrease of the nasopharyngeal mass, with residual but improved enhancement of the skull base and clivus (Figure 4). Her acute radiation-induced side effects had largely resolved. Her clinical and radiographic exam remained stable for two years. Unfortunately, at two years post-treatment, she developed a progressive nodular enhancement within the left nasopharynx (treated within the previous high dose region), which was subsequently salvaged with stereotactic photon radiotherapy (4500 cGy in five fractions, delivered every other day). She had no evidence of Grade 3 or higher late toxicity of radiotherapy at the time of her salvage treatment.

## Discussion

This case posed a unique treatment planning challenge, as the patient’s ACC was deemed unresectable due to its location in the nasopharynx and its proximity to the critical neural structures; thus, she was treated with a radiation treatment plan utilizing proton beam therapy. ACC accounts for only 0.5% to four percent of all carcinomas of the nasopharynx [1,3]. Due to ACC’s rare occurrence in this location, there is limited evidence and no consensus with regards to optimal management. Local control is important, as ACC tends to recur and invade local and adjacent structures, particularly nerves, causing significant morbidity and increased risk of mortality to the patient. Of the reported case series available to date, the standard of care with the highest local control typically involves upfront surgery followed by radiotherapy [2,4-5]. In circumstances where tumor invasion and/or location precludes definitive surgical management, definitive radiotherapy may be offered as an alternative treatment option to achieve local control [2,4-6].

Of the potential radiotherapy treatment options available for this case, proton beam radiation was selected as the mainstay of therapy due to the unique advantage it offers in regards to the treatment of nasopharyngeal ACC located in unresectable regions. As ACC has a predilection for perineural invasion and tracking, our target volumes encompassed the adjacent cranial nerves to the skull base foramens, particularly the foramen ovale and the jugular foramen. Liu et al., in a study of 26 patients with nasopharyngeal ACC, reported that cranial nerve invasion, advanced-stage disease, and nonsurgical treatments were poor prognostic factors associated with poor overall survival [2]. In our case, an IMRT plan would have required a reduction in dose coverage of the target that was adjacent to radiosensitive neural structures (such as the optic nerves and brain) in order to meet the safe dose tolerance constraints for these structures. An IMPT plan, however, allowed for a conformal high dose target coverage with maximum sparing of the adjacent normal tissue while maintaining high dose concentration to the intended target [4,7-8]. Given the physical properties of the proton beam, most of its energy is deposited at the target with minimal exit dose, thereby reducing the dose to the adjacent brain and optic apparatus [7-8]. In addition, there is a perceived higher radiobiological effectiveness of protons compared to photon therapy due to the proton’s higher linear energy transfer properties and, therefore, the potentially higher cell kill [9].

Due to the rare occurrence of ACC in the nasopharynx, there is a limited amount of literature regarding proton therapy as a form of treatment in this setting. Studies that had been conducted to date indicate that proton beam therapy resulted in promising local control for patients with unresectable ACC of the nasopharynx. Gentile et al. reported long-term outcomes in 14 patients with unresectable ACC of the nasopharynx treated with proton therapy with a median follow-up of 69 months [4]. The five-year overall survival was 59% and only three patients developed Grade 3 or higher late toxicity. In addition, results from MD Anderson Cancer Center recorded the responses of eight patients with unresectable head and neck ACC treated with IMPT: four patients achieved complete response, four patients had stable diseases, and only one patient developed late Grade 4 optic nerve toxicity. Taken together, these studies highlight the potential effectiveness of IMPT in treating unresectable ACC with limited long-term toxicity [10]. 

While there are case series indicating promising results for local control when utilizing radiation therapy as the mainstay of treatment for unresectable ACC, the utility of proton beam therapy in this subgroup of patients remains a question and longer-term data is required to compare the effectiveness and late toxicity profile of proton beam therapy compared to photon therapy.

## Conclusions

In this report, we presented a case of unresectable, locally advanced ACC of the nasopharynx treated with IMPT. The patient ultimately achieved a good two-year local control with no significant late toxicity. At her two-year follow-up visit, the patient was found to have a recurrence. While proton beam therapy has previously resulted in promising local control for patients with unresectable ACC of the nasopharynx, further follow-up is needed to assess the durability of response given the long natural history of the disease.
